# Epidermal Wnt signalling regulates transcriptome heterogeneity and proliferative fate in neighbouring cells

**DOI:** 10.1186/s13059-017-1384-y

**Published:** 2018-01-15

**Authors:** Arsham Ghahramani, Giacomo Donati, Nicholas M. Luscombe, Fiona M. Watt

**Affiliations:** 1 The Francis Crick Institute, 1 Midland Road, London, NW1 1AT UK; 2 King’s College London, Centre for Stem Cells and Regenerative Medicine, 28th Floor, Tower Wing, Guy’s Hospital, Great Maze Pond, London, SE1 9RT UK; 3 Department of Life Sciences and Systems Biology, University of Turin, Via Accademia Albertina 13, 10123 Torino, Italy; 4 UCL Genetics Institute, University College London, London, WC1E 6BT UK; 5 Okinawa Institute of Science & Technology Graduate University, Okinawa, 904-0495 Japan

## Abstract

**Background:**

Canonical Wnt/beta-catenin signalling regulates self-renewal and lineage selection within the mammalian epidermis. Although the transcriptional response of keratinocytes that receive a Wnt signal is well characterized, little is known about the mechanism by which keratinocytes in proximity to the Wnt-receiving cell are co-opted to undergo a change in cell fate.

**Results:**

To address this, we perform single-cell RNA-sequencing on mouse keratinocytes co-cultured with and without beta-catenin-activated neighbouring cells. We identify five distinct cell states in cultures that had not been exposed to the beta-catenin stimulus and show that the stimulus redistributes wild-type subpopulation proportions. Using temporal single-cell analysis, we reconstruct the cell fate change induced by Wnt activation from neighbouring cells. Gene expression heterogeneity is reduced in neighbouring cells and this effect is most dramatic for protein synthesis-associated genes. Changes in gene expression are accompanied by a shift to a more proliferative stem cell state. By integrating imaging and reconstructed sequential gene expression changes during the state transition we identify transcription factors, including Smad4 and Bcl3, that are responsible for effecting the transition in a contact-dependent manner.

**Conclusions:**

Our data indicate that non-cell autonomous Wnt/beta-catenin signalling decreases transcriptional heterogeneity. This furthers our understanding of how epidermal Wnt signalling orchestrates regeneration and self-renewal.

**Electronic supplementary material:**

The online version of this article (doi:10.1186/s13059-017-1384-y) contains supplementary material, which is available to authorized users.

## Background

The mammalian epidermis comprises interfollicular epidermis (IFE), hair follicles, sebaceous glands and sweat glands. Under steady-state conditions, each of these compartments is maintained by distinct populations of stem cells. However, following wounding, each stem cell subpopulation exhibits the capacity to contribute to all differentiated lineages [1]. Recent single-cell gene expression profiling of adult mouse epidermis identified multiple epidermal subpopulations [2]. Furthermore, in cultures of human and mouse keratinocytes, there are three or more subpopulations with varying proliferative potential [3, 4].

One pathway that plays a key role in regulating stem cell renewal and lineage selection in mammalian epidermis is Wnt/beta-catenin signalling, which is an important regulator of epidermal maintenance, wound repair and tumorigenesis [5, 6]. Gene expression profiling has identified a number of signalling pathways that are regulated by cell-intrinsic activation of beta-catenin. Wnt signalling is indispensable for adult epidermal homeostasis; loss of beta-catenin in the IFE causes a defect in stem-cell activation, resulting in reduced basal layer proliferation and IFE thinning [7, 8], and loss of hair follicles. Conversely, transient activation of epidermal beta-catenin in adult epidermis leads to expansion of the stem-cell compartment and results in the formation of ectopic hair follicles, at the expense of the sebaceous glands, and an increase in IFE thickness [9, 10].

There is good evidence that intrinsic beta-catenin activation in epidermal keratinocytes leads to effects on neighbouring epidermal cells. For example, in the mouse hair follicle, activated mutant beta-catenin cells can co-opt wild-type (WT) cells to form a new hair growth through secretion of Wnt ligands [9, 11]. This form of non-cell autonomous (NCA) activation suggests that autonomous Wnt signalling has the capability to change neighbouring cell fate. Although the mechanisms of autonomous Wnt activation are well described, it is unclear how NCA effects differ from cell intrinsic effects and how beta-catenin can simultaneously regulate self-renewal while changing the fate of neighbouring cells.

In this study, we set out to analyse NCA signalling in WT mouse keratinocytes that were co-cultured with keratinocytes in which beta-catenin was activated. This has revealed previously unknown heterogeneity of WT mouse keratinocytes and elucidated the effect of Wnt signalling on neighbouring cell state and heterogeneity.

## Results

### Single-cell messenger RNA-sequencing analysis of basal epidermal stem cells

To explore the effects of non-cell autonomous Wnt signalling on epidermal cell state we sequenced the transcriptomes of single WT murine keratinocytes co-cultured with cells expressing an inducible form of stabilised beta-catenin (K14ΔNβ-cateninER) in a ratio of 9:1. We compared cells cultured in the absence of 4-hydroxy-tamoxifen (4OHT) with cells treated for 24 h with tamoxifen to induce beta-catenin. Cells were then disaggregated, loaded onto the C1 96-well microfluidic device (Fluidigm) and captured for sequencing. Owing to the single-cell capture method used, highly keratinized and terminally differentiated cells > 20 μm in diameter were excluded. We identified K14ΔNβ-cateninER cells by aligning reads to the transgene sequence and subsequently removed these cells from analysis (ten untreated cells and 14 activated cells). After quality control, we retained 125 WT control cells and 129 WT cells exposed to Wnt signalling neighbours. We recorded a median of 641,000 reads per cell equating to 4000–8000 genes expressed per cell (transcripts per million [TPM] > 1). Read alignment distribution was in line with other single-cell RNA-sequencing (RNA-seq) datasets with minimal ribosomal and intergenic reads (Additional file 1: Figure S1).

**Fig. 1:**
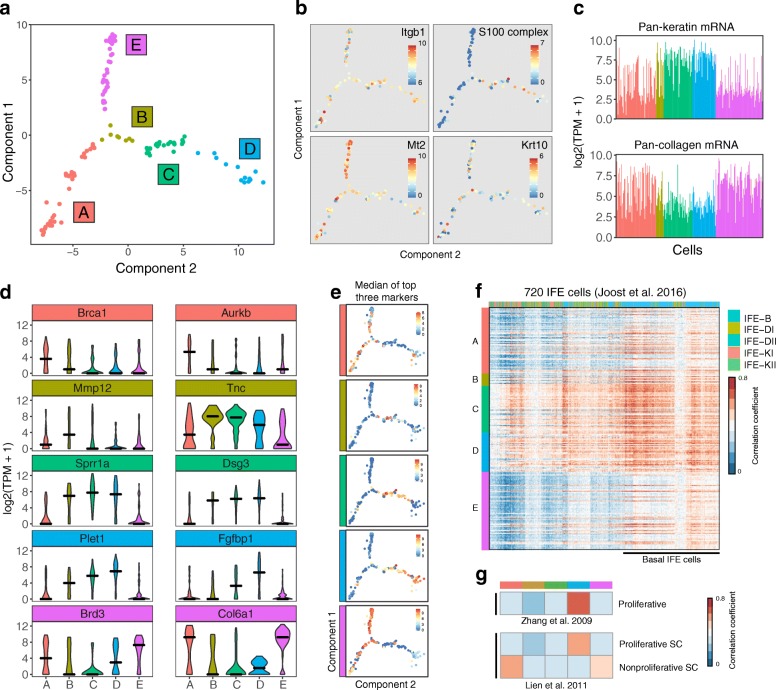
Molecular heterogeneity of epidermal cells in culture. **a** Epidermal cell transcriptomes and cell state relation visualised using DDRTree and coloured according to unsupervised clustering. Each data point is one cell and axes are dimensionally reduced components of the cell transcriptomes. *Colours* and *numbers* represent the five identified cell states. **b** Gene expression for four marker genes shown for each cell on the state map. *Top left*: Integrin beta-1 (Itgb1), a basal IFE marker. *Top right*: S100 differentiation associated genes. *Bottom left*: Mt2, a basal IFE marker. *Bottom right*: Keratin 10, a suprabasal IFE marker of commitment to differentiation. **c** *Barplot* showing mean expression per cell averaged over all keratin associated messenger RNAs (mRNA) (*top*) and all collagen mRNAs (*bottom*). **d** Distribution of the expression of cluster-specific markers *coloured* according to cell state. Two markers are shown per cell state. *Black line* represents the median expression of the cluster. **e** Median expression of the top three markers for each state per cell on the state map. **f** *Heatmap* showing the similarity between epidermal cells from this study and IFE cells from Joost et al. Similarity is measured by Pearson’s correlation coefficient. Cells from this study are *coloured* by cluster along the *vertical axis*. Cells from Joost et al. are *coloured* by differentiation status along the *horizontal axis*. Joost et al. IFE cell legend is shown in order of differentiation status. B basal IFE cells, DI/DII differentiated suprabasal IFE cells, KI/KII keratinized IFE cells. **g** *Heatmap* showing similarity between cluster average transcriptomes from this study and proliferative IFE cells from Zhang et al. [14] and activated vs quiescent IFE cells from Lien et al. [15]

To explore cell-state heterogeneity in WT keratinocytes that had not been exposed to a neighbour in which beta-catenin was activated (untreated samples), we used reverse graph-embedding, a machine-learning technique. This enabled us to reconstruct a parsimonious tree connecting all observed epidermal cell states (DDRTree, Monocle 2) [12]. We applied the DDRTree algorithm to WT cells using expressed genes (TPM > 1) after removing cell-cycle associated genes. We identified five distinct in vitro cell states (Fig. 1a) forming three major branches that represent varying states of proliferation and differentiation.

States A and E showed highest expression of Mt2, a basal IFE marker, alongside markedly low expression of S100 epidermal differentiation complex genes in comparison with the remaining subpopulations. These expression patterns indicate states A and E represent transcriptomic signatures before commitment to differentiation (Fig. 1b; top left) [13]. Itgb1, another marker of basal IFE cells, showed variable expression in vitro in comparison to Mt2, but was expressed in all cells (Fig. 1b; top right)*.* The differentiation marker Krt10 was variably expressed across all subgroups (Fig. 1b; bottom right) [4]. Separation between pre- and post-commitment cell states is further apparent when looking at pan-keratin and pan-collagen gene expression. In vivo, keratinocytes commit to differentiation upon detaching from the basement membrane, resulting in reduced collagen expression and increase in overall keratin content. States B, C and D express significantly more keratin messenger RNAs (mRNA) and conversely states A and E are characterised by higher collagen mRNA levels (Fig. 1c; Kolmogorov–Smirnov test, *p* < 0.05). For each cell state, we determined genes differentially expressed vs the remainder of the population and identified between six (state B) and 101 (state C) markers (Fig. 1d, Additional file 2: Table S1). We observed that the median expression of the top three markers for each state was sufficient to distinguish each state (Fig. 1e).

To determine the putative biological function of each cell state, we correlated the single-cell signatures with a comprehensive set of IFE subpopulation expression profiles identified by Joost et al. We further integrated two other bulk gene expression studies which identified signatures for proliferative and non-proliferative epidermal stem cells [14, 15]. From comparison with the Joost IFE subpopulations, all of our single cells correlate strongly with basal IFE stem cells, as expected since large (>20 μm) terminally differentiated cells were excluded from the analysis (Fig. 1f). States A, and to a lesser extent state E, showed high correlation with an isolated subpopulation of ‘non-proliferative self-renewing’ epidermal cells characterised by Lien et al., which we would describe, for simplicity, as non-proliferative stem cells. (Fig. 1g). We concluded that states A and E both exhibit a self-renewing cell gene expression signature but differ in proliferative state. States B, C and D formed a branch of the cell trajectory representing early commitment to differentiation, characterized by expression of proliferation associated genes from Roshan (e.g. MRPL33, YY1) and correlated strongly with the expression profile of proliferative keratinocytes from Zhang [3, 14]. This branch of the state tree shows expression of early differentiation markers such as MXD1, Dsc2, Dsg3 [16] and highest expression of S100 early differentiation-associated genes. Figure 1a summarises the state classification of cells as determined by our cluster and DDRTree analysis, highlighting the relationship between our three branches composed of five identified keratinocyte states.

### Inducible Wnt signalling

**Fig. 2:**
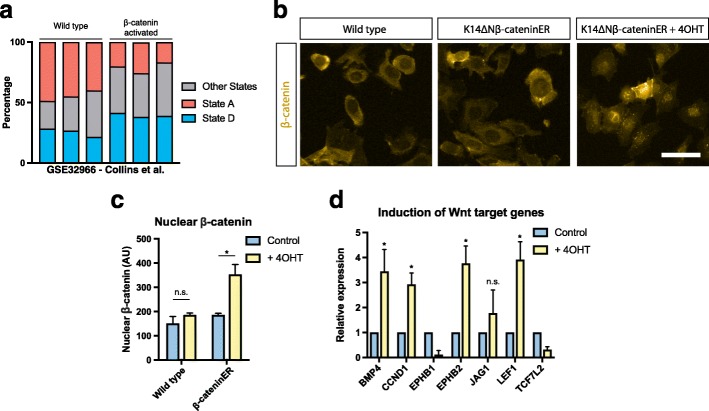
Induction of canonical Wnt signalling in a subpopulation of cells. **a** Estimated proportions of state A and state D cells in WT and beta-catenin activated epidermis samples from Collins et al. (GSE32966) [17]. **b** Immunofluorescence showing cytoplasmic and nuclear beta-catenin in WT and K14ΔNβ-cateninER keratinocytes after induction of canonical Wnt signalling using 4OHT. Scale bar, 20 μm. **c** Quantification of mean nuclear beta-catenin fluorescence intensity (n = 3 independent cultures). **d** Activation of canonical Wnt target genes in K14ΔNβ-cateninER cells upon induction with 4OHT quantified by quantitative reverse transcription polymerase chain reaction (n = 4). *P < 0.05, n.s. not significant. All data shown as mean ± SD

In a previous study, we generated gene expression profiles from WT and beta-catenin activated adult mouse epidermis [17]. We reanalysed these data to estimate the relative proportion of cells in each of the cell states identified in vitro (Fig. 1a). We utilised CIBERSORT, a method for characterising the composition of tissue expression profiles resulting from mixtures of cells [18]. Our reanalysis indicated that epidermal beta-catenin signalling results in a depletion of cells in state A and increases the abundance of cells in state D (Fig. 2a). This is consistent with the in vivo observation that intrinsic activation of epidermal beta-catenin results in proliferation and expansion of the stem cell compartment [10].

Next, to investigate whether epidermal cell states were altered by NCA Wnt signalling, we examined the treated sample, WT keratinocytes co-cultured with K14ΔNβ-cateninER keratinocytes in the presence 4OHT. We confirmed that K14ΔNβ-cateninER cells intrinsically activated canonical Wnt signalling in response to 4OHT by detecting beta-cateninER translocation into the nucleus (Fig. 2b, c; Lo Celso et al. [10]). We also validated upregulation of canonical downstream target genes such as Bmp4, Cyclin-D1 and Lef1 in K14ΔNβ-cateninER keratinocytes using quantitative reverse transcription polymerase chain reaction (qRT-PCR) (Fig. 2d).

### Reconstruction of NCA Wnt induced state transition

**Fig. 3:**
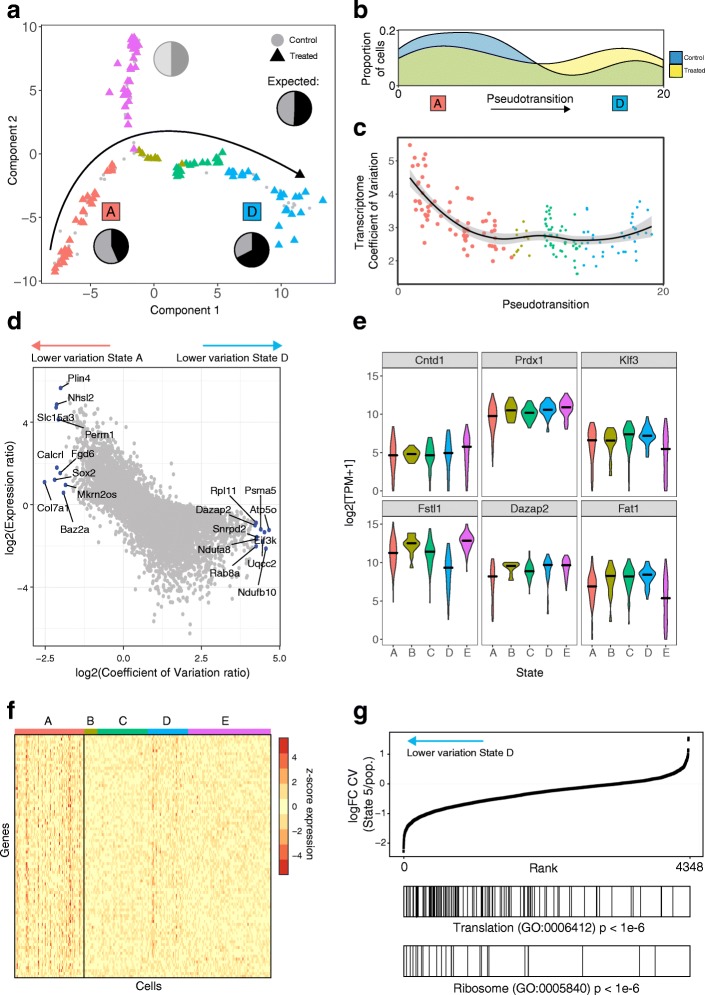
Neighbouring Wnt activation reduces gene expression heterogeneity of protein-synthesis associated genes. **a** Keratinocytes exposed to Wnt signalling neighbours projected onto the WT epidermal cell state map. WT cells shown with *grey circles*, exposed cells shown *coloured* by cell cluster as *triangles. Pie charts* show relative proportions of WT vs signalling-exposed cells in each cell state cluster. States A and D are labelled as these states contain significantly different proportions of exposed vs unexposed cells. **b** Density of control vs NCA Wnt exposed cells along the reconstructed pseudotransition from states A to D. **c** Transcriptome coefficient of variation per-cell (TCOV), *coloured* by cell state and point size represents number of genes detected as expressed (TPM > 1) for that cell. *Line* depicts a loess-curve fit for the pseudotransition–TCOV relationship. **d** *Scatterplot* showing the log-ratio of coefficient of variation vs the log-ratio of gene expression between states A and D. Genes labelled depict the ten highest and lowest coefficient of variation ratios. Also see Additional file 1: Figure S2 for comparisons between states A and B and states B and D. **e** *Violin plots* depicting expression of cluster-specific genes showing differential dispersion between states. **f** *Heatmap* with symmetric gene expression scale (z-score normalised log2(TPM + 1)) showing reduction in gene expression heterogeneity of 281 genes between state A and the remaining transition states. **g** Gene rank enrichment analysis of log-fold change in gene coefficient of variation (CV) between cells in state D and the remainder of the cell population. Genes with lower variation in state 5 are enriched for translation and ribosome-associated gene ontology (GO) annotations (*p* value < 1e-6)

Having identified several different states of WT keratinocytes and validated the intrinsic effects of beta-catenin activation, we used single-cell RNA-seq to deconvolve the effects of NCA Wnt signalling. Single-cell transcriptomes from WT keratinocytes co-cultured with 4OHT-activated K14ΔNβ-cateninER cells were compared with those of WT cells co-cultured with uninduced K14ΔNβ-cateninER cells and mapped onto the same dimensionally reduced space (Fig. 3a). To exclude the possibility of transcriptional changes resulting from 4OHT treatment alone, we screened bulk differential gene expression between the two cohorts of WT cells. We found no evidence of oestrogen receptor target genes among differentially expressed genes [19].

We observed the same five distinct transcriptional states in WT cells with or without NCA Wnt signalling (Fig. 3a), confirmed by independently analysing the treated cell population to reveal five equivalent subpopulations. However, exposure to non-cell autonomous Wnt signalling markedly changed the state distribution of keratinocytes (Fisher’s exact test, *p* < 0.05). Pie charts in Fig. 3a show the observed ratio of control and signalling-exposed cells. States A and D significantly deviated from the expected ratio (binomial test, *p* < 0.05). After exposure to NCA signalling, there was a depletion of cells in the non-proliferative stem cell state A and a higher than expected proportion of cells in state D, representing a transition towards a proliferative and more differentiated transcriptional state (Fig. 3a).

Taking the states with altered cell proportions and the transition states in between (states A, B, C and D), we reconstructed the state transition induced by neighbouring Wnt + keratinocytes using the Monocle pseudotime method [12]. WT and exposed cells were ordered from state A to state D to reconstruct the temporal order of gene expression changes for cells undergoing this transition, referred to as the pseudotransition. Figure 3b shows the proportion of control and Wnt signalling exposed cells along the reconstructed temporal transition from state A to state D; from this distribution it is clear that NCA Wnt exposed cells bias towards State D.

### NCA Wnt signalling reduces heterogeneity in protein synthesis-associated transcripts

We next sought to understand why state A cells were uniquely depleted after neighbour Wnt signalling. Previous studies have shown that cell responses to extrinsic signalling are affected by intracellular and intercellular transcriptional noise [20–22]. We thus hypothesised that the response to NCA Wnt signalling involves changes in both the dynamic range of transcriptional variation (intracellular variation) and state-specific gene expression (intercellular variation).

We first examined whether there was a difference in intracellular transcriptomic heterogeneity between the three altered states and whether changes occurred along the pseudotransition. The resulting ordering of cells from state A to state D was used to examine the transcriptome coefficient of variation (TCOV) per cell (Fig. 3c). Here, TCOV is an intracellular measure of the spread of transcript abundance accounting for mean abundance. Notably, TCOV decreased over the state transition and was significantly higher in state A than states B, C and D (Kolmogorov–Smirnov test; *p* value < 0.05). This reduction in dynamic range of gene expression is consistent with previous studies that have shown that progenitor cells have a higher rate of stochastic multilineage gene expression that reduces upon cell-fate commitment [23, 24].

Next, we contrasted the heterogeneity of genes that do not change in expression level between the transition states (Additional file 1: Figure S2A–C). Figure 3d displays the relationship for the log-ratio of intercellular gene expression variation and expression level between the two extremes of the pseudotransition, states A and D, with the top ten differentially dispersed genes labelled. Of interest are genes that change in expression heterogeneity from state A to state D while remaining at constant expression levels. Notably, Baz2a, Sox2, Col7a1 and Calcrl were among the genes with reduced COV in state A without significant differential expression (Additional file 2: Table S2). Baz2a has been previously established as part of the nucleolar remodelling complex that is important for establishing epigenetic silencing and transcriptional repression of ribosomal RNA genes [25, 26]. Sox2 is an adult stem cell factor shown to be expressed in multiple epithelia [27]. Sox2 has been previously reported to be expressed in hair follicles but absent from the interfollicular epidermis [28]. Similarly, Col7a1 and Calcrl are significantly upregulated in hair follicle bulge stem cells [29]. Additional file 2: Table S2 lists the statistically significant and differentially heterogeneous genes characteristic of each state.

Figure 3e shows the expression variability for a selection of statistically significant (q < 0.05) differentially heterogeneous genes. We found multiple patterns of intercellular heterogeneity including genes that differed in variability across branches and genes that were selectively more variable in a single state. Peroxiredoxin-1 (Prdx1) is one such gene, which showed strongly variable expression in state A in comparison to the remaining states despite no statistically significant difference in median expression level.

When the four transition states were compared, we observed many more heterogeneously expressed genes in state A than states B, C or D. The contrast in number of differentially dispersed genes is demonstrated using the symmetric expression scale in Fig. 3f. We observed 281 significant and differentially heterogeneously expressed genes in state A, compared with only 19 genes in state D. A striking number of the heterogeneously expressed genes in state D are known regulators of stem cell identity such as Cdh11, Cav2 and Apc [30–32]. They are typically upregulated in basal stem cells; however, little is known about how their heterogeneity affects cell fate. Our analysis of intercellular variation suggests that in stem cells (state A) with low RNA and protein metabolism [33], transcriptional heterogeneity is lowest for stem-cell marker genes, emphasising the importance of transcriptional noise in addition to transcriptional amplitude. These observations are consistent with our hypothesis that state A cells are responsive to NCA Wnt signalling due to greater transcriptional variability. Exposure to this coordinated extracellular stimulus reduces transcriptional heterogeneity for these cells and biases their fate towards state D.

To determine genes essential for a cell to be receptive to neighbour Wnt activation, we analysed the fold-change in heterogeneity between states A and D, comprising the majority of genes with differential heterogeneity. We found strong enrichment for translation and ribosome-related genes, indicating a role for protein synthesis (*p* < 1e-6, Fig. 3g). We hypothesise that cells in state A exhibit a multilineage primed transcriptional programme with stochastic expression of metabolism associated genes. Upon fate commitment, cells in the IFE steadily increase their translational rate in a proliferation independent manner [33]. Hence, translation associated genes are subject to greater transcriptional regulation post commitment, independent of transcription level.

These data and our single-cell analysis identified an NCA Wnt-receptive subpopulation, state A, with greater dynamic range in gene expression (TCOV) and greater variation in the abundance of protein synthesis associated transcripts. Introduction of the NCA Wnt stimulus reduces variability in both aspects.

### Transcription factors driving cell fate change

**Fig. 4:**
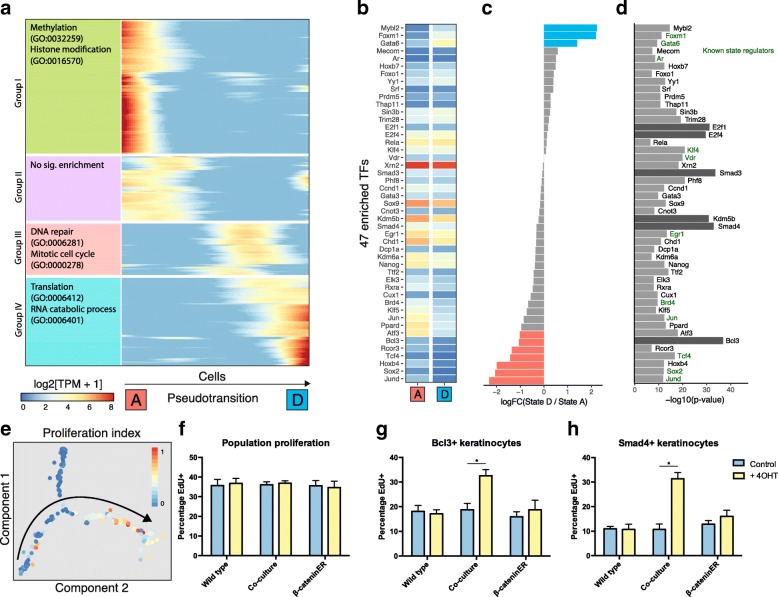
Induction of proliferation in Wnt + neighbours is regulated by 47 TFs. **a** *Heatmap* showing smoothed expression of pseudotransition-dependent genes (n = 632) ordered by hierarchical clustering and maximum expression. Top two enriched GO terms shown on *left* (all significant at q < 0.05). Genes (*rows*) are ordered by peak expression from state A to state D. **b**–**d** *Heatmap* showing expression of 47 TFs enriched as regulators of pseudotransition-dependent genes (**b**). Log-fold change in expression of TFs between state D and state A to show strongly directional TFs (**c**) and enrichment level of TFs (**d**). **e** Normalised proliferation index projected onto the cell state map. *Arrow* denotes the direction of pseudotransition. **f**–**h** Quantification of population proliferation by EdU assay in WT, K14ΔNβ-cateninER and co-cultured keratinocytes with and without 4OHT treatment (**f**). After stratification by Bcl3 nuclear abundance, cells in co-culture with activated K14ΔNβ-cateninER cells show a relative higher proliferation rate (**g**). Similarly, stratification by nuclear Smad4 shows higher proliferation in the treated co-culture condition (**h**) (n = 3 independent cultures). *P < 0.05. All data shown as mean ± SD

To understand drivers of the observed differential heterogeneity, we reconstructed transcriptional changes over time along the state trajectory. Expression of each gene was modelled as a non-linear function of pseudotransition time [12]. We found 632 genes that were dynamically regulated over the state transition (false discovery rate < 5%; Fig. 4a). Using hierarchical clustering, we grouped these genes into four patterns of dynamic expression. Group I genes, most highly expressed in state A, were enriched for methylation associated genes and histone modifiers such as Setd3 and Kdm7a. These genes represent a pre-transition transcriptional profile of state A cells without exposure to signalling beta-catenin induced cells. Group III genes show highest expression in state C, an intermediate transition state, with enrichment for desmosome genes such as Dsc2, Dsc3, Dsg2 and Dsp, which are most highly expressed in the suprabasal layers of murine epidermis indicative of early commitment to differentiation [2]. Group IV genes, predominantly expressed in state D, were enriched for protein synthesis associated genes and entry into mitosis, respectively. Notably, group III includes the transcription factor (TF) Klf5, a regulator of proliferation in intestinal epithelial cells [34], and E2f1, which leads to epidermal hyperplasia when overexpressed in mice [35].

To gain insight into the regulation of the dynamically expressed genes induced by Wnt + cells, we performed a TF motif analysis (Fig. 4b–d). We calculated enrichment of TF binding sites from the ChEA database, removing TFs that were not expressed in any of the five cell states (log[TPM + 1] > 1). By analysing the promoters of the 632 dynamically expressed genes, we identified 47 TFs putatively regulating the state transition. TFs were separated into three groups according to the directionality of gene expression from state A to state D: positive; negative; and neutral. We noted that the activities of some identified TFs such as Smad3 and Smad4 are only partially dependent on expression level. Hence, we did not rule out TFs on the basis of expression.

From this analysis we predicted Smad3, Smad4, Kdm5b, E2f1 and E2f4 as previously unknown key regulators of the state transition, with Bcl3 as a likely regulator of the specific transition between states A and D. Known regulators of keratinocyte cell fate are shown in blue (Fig. 4d). Of note are Gata6 and Foxm1, two TFs upregulated in state D and previously shown to mark cells with multi-lineage differentiation potential and increased proliferative capacity, respectively [36–38]. We hypothesised that epidermal cells could be stratified based on the nuclear abundance of our identified novel state-regulating TFs, specifically Smad4 and Bcl3. Furthermore, our analysis on state D gene expression markers and transcriptome correlation indicated that this state shows a higher proliferation rate relative to states A and B. To investigate further, we calculated a cell proliferation index consisting of normalised expression of S-phase markers (Fig. 4e). This index demonstrated that states C and D comprise proliferative cells, with few cells in states A, B or E actively proliferating.

To confirm our findings, we used an EdU incorporation assay to distinguish proliferating cells and analysed whether keratinocytes positive for our predicted driver TFs (measured by nuclear intensity) were more likely to be proliferative. At the population level, there was no significant difference in proliferation when epidermal cells were co-cultured with 4OHT induced K14ΔNβ-cateninER cells (Fig. 4f). However, when cells were discriminated by nuclear intensity for Bcl3 or Smad4 we observed a significant difference between WT cells exposed to NCA Wnt signalling and WT or induced K14ΔNβ-cateninER cells alone (Fig. 4g and h). On average, 18% of Bcl3^+^ cells were positive for EdU uptake in WT or uninduced K14ΔNβ-cateninER cells; however, when WT cells were co-cultured with induced K14ΔNβ-cateninER cells the EdU^+^ fraction rose to 34%. The proportion of proliferative Smad4^+^ cells increased from 10–18% to 33% EdU^+^.

Taken together these results indicate that Bcl3 and Smad4 are specific markers of the epidermal state transition and mark cells moving along the trajectory between state A and state D (Fig. 1) during the first 24 h of exposure to a NCA Wnt signal.

### NCA Wnt induced state transition is contact dependent

From our total population of 129 cells exposed to NCA Wnt signalling, cells in state E appeared to be unaffected. Our data suggest that state A comprises cells in a ‘responder’ state permissive to NCA Wnt signalling due to the presence of key TFs and a more heterogeneous gene expression programme. We sought to address whether the reduction in ribosome-related gene expression heterogeneity and the induced expression of transition TFs are contact- or distance-dependent. To answer this question, we labelled co-cultures of WT and K14ΔNβ-cateninER cells with a cell reporter of protein synthesis.

**Fig. 5:**
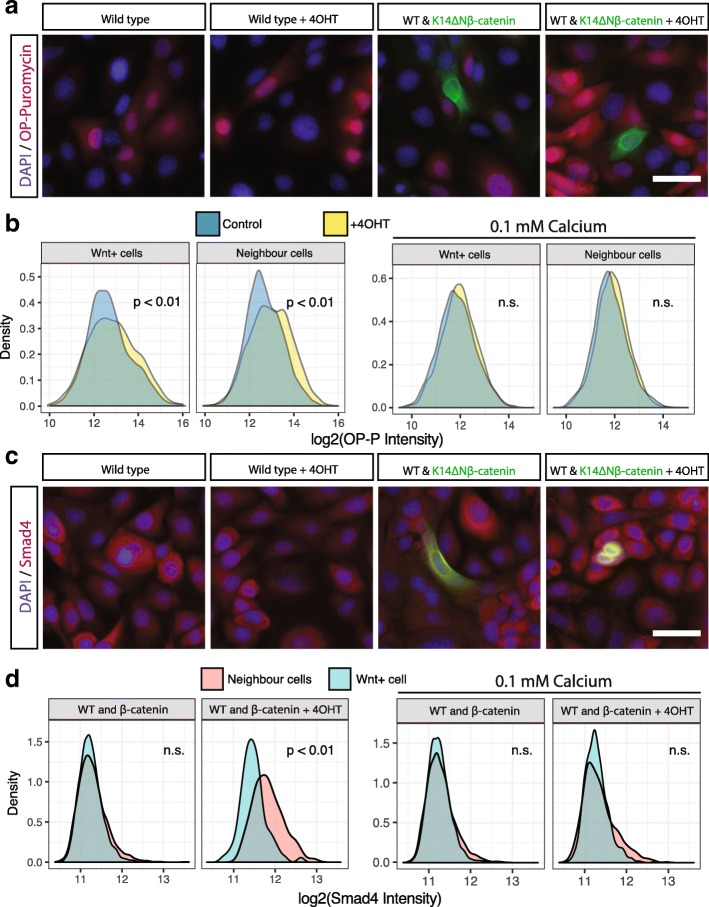
Wnt + induced cell state transition is contact-dependent. **a** Appearance of typical WT cells and K14ΔNβ-cateninER co-cultures stained for OPP. **b** *Left*: Quantification of OPP in K14ΔNβ-cateninER cells and their neighbours in control and 4OHT treated conditions. *Right*: Similar quantification for cells co-cultured in low calcium medium (0.1 mM) to inhibit cell–cell contact (*n* = 3 independent cultures, *n* = 3 technical replicates, all pooled). **c** Appearance of typical WT cells and K14ΔNβ-cateninER co-cultures stained for Smad4. **d** *Left*: Quantification of Smad4 in K14ΔNβ-cateninER cells and their neighbours in control and 4OHT treated conditions. *Right*: Similar quantification for cells co-cultured in low calcium medium (0.1 mM) to inhibit cell–cell contact (*n* = 3 independent cultures, *n* = 3 technical replicates, all pooled). All scale bars, 20 μm, n.s. not significant

We measured global protein translation by assaying incorporation of O-propargyl-puromycin (OPP) and compared WT cells in contact with induced K14ΔNβ-cateninER cells to untreated control cells (Fig. 5a). We observed that WT cells showed higher translational activity when in contact with a Wnt + cell. To confirm this, we analysed the neighbours of over 10,000 Wnt + cells and compared the OPP fluorescence intensity distributions (Fig. 5b). We found a small but statistically significant increase in translation rate for both Wnt + cells and neighbour cells in the 4OHT treated condition. This suggested a contact-dependent mechanism for control of protein synthesis downstream of NCA Wnt signalling.

To confirm contact dependence and to rule out local diffusion of soluble factors, we repeated the assay in low calcium conditions. Keratinocytes cultured in low calcium medium do not form adherens or desmosomal cell contacts [39, 40]. Strikingly we observed no NCA Wnt effect under these conditions (Fig. 5b). Similarly, we observed no increase in nuclear abundance of Smad4, our predicted TF downstream of NCA Wnt signalling, in Wnt + cells, as predicted. However, in neighbouring cells there was a significant increase in nuclear Smad4 intensity, which is abrogated in low calcium conditions (Fig. 5c and d).

Taken together these data suggest that the Smad4-mediated cell state transition is downstream of non-cell autonomous Wnt signalling. Furthermore, the induction of this transition is contact dependent and does not occur under conditions where desmosomal adherens junctions are inhibited.

## Discussion

Heterogeneity in the self-renewal and proliferative capabilities of keratinocytes has long been recognised. Previous analysis of clones and subclones of cultured human epidermal cells has demonstrated that there are at least three subpopulations, ‘holoclones’, ‘meroclones’ and ‘paraclones’ with descending self-renewal potential [4, 41, 42]. More recently, Roshan et al. have shown the existence of two in vitro states with differing proliferation rates and single cell transcriptomics have identified two distinct subpopulations of human keratinocytes in culture [31]. In this study, we have dissected molecular heterogeneity of epidermal cells at greater resolution and extended previous research by exploring the response of keratinocytes to neighbouring cells in which beta-catenin is activated. We have identified five distinct transcriptomic states and characterised their biological relevance in order to create a state map of keratinocytes in vitro.

**Fig. 6:**
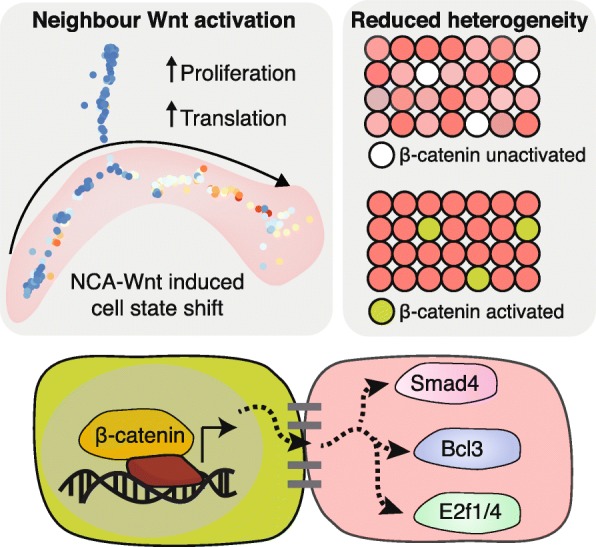
Graphical abstract - Wnt/beta-catenin activation affects neighbouring cells. Epidermal cells in culture adopt one of five distinct transcriptomic states differing on the basis of proliferation and commitment to differentiation. Wnt/beta-catenin signalling acts as a non-cell autonomous signalling cue to activate a handful of TFs including Smad3/4, E2f1/4 and Bcl3 in neighbours. Concurrently, transcriptional heterogeneity is reduced as neighbour cells enter into a committed and proliferative transcriptional state

Using the cell state map and inducible activation, we have shown that Wnt/beta-catenin signalling acts to perturb cell fate by co-opting neighbours to become biased towards a pre-existing proliferative fate (Fig. 6). It is important to note that we found no evidence for a de novo cell state as a result of non-cell autonomous signalling. This highlights the relevance of transient Wnt/beta-catenin signalling to cell state and is consistent with a model of stochastic epidermal commitment where extrinsic cues alter the likelihood of a cell switching state [43]. The observed difference in transcriptome variability between states A and D reflects a difference in cell state stability. Only a modest increase in translational activity is observed in state D or neighbouring cells; however, there is a marked reduction in the variability of translation-associated genes, highlighting the importance of transcriptional noise as well as transcriptional volume for determination of cell state.

Combined transcriptomic and positional single cell analyses allowed us to resolve spatial and temporal effects. As a result of this, we identified a collection of TFs, many of which were not previously implicated in epidermal cell state. One example is Bcl3, which is expressed in murine and human basal IFE; however, its role in epidermal cell fate is poorly understood [2, 44]. In addition, we identified Smad4 and utilised this as a marker of cell state transition. Smad4-beta-catenin cross-talk has been previously identified as essential for hair follicle maintenance [45–47]. Here, we show that beta-catenin signalling activation leads to Smad4 activation in a non-cell autonomous manner.

Our study does not address the extracellular effectors of NCA Wnt signalling. We previously identified a diverse array of secreted signalling molecules downstream of canonical Wnt signalling, including Bmp6, Dkk3, Wnt ligands, cytokines and ECM components [48]. Our analysis of TF effectors and previous evidence of epidermal self-renewal via autocrine Wnt signalling suggests that cells neighbouring a beta-catenin^+^ cell are simultaneously committed to a state of lesser self-renewal and greater proliferative abilities. This is achieved via a combination of Bmp signalling (effected through Smad3/4; Fig. 6) and neighbouring Wnt inhibition (Dkk3, Lim et al.). Intriguingly these effects are contact-dependent, hinting at yet-unknown mechanisms of locally restricting these signalling molecules or major signalling contributions from other membrane-bound factors. The observed difference in translation rate and proliferation in neighbouring cells demonstrates asymmetric coupling of cell fates, an essential component of epidermal homeostasis to ensure a balance of cell fates and epidermal metabolism.

## Conclusions

In conclusion, our data provide a framework for studying cell state in the interfollicular epidermis and extend our understanding of functional heterogeneity and NCA signalling. Using this knowledge, we demonstrate how Wnt/beta-catenin signalling, an orchestrator of regeneration, homeostasis and tumorigenesis in multiple tissues, influences neighbouring cell fate.

## Methods

### Cell isolation and culture

K14ΔNβ-cateninER transgenic mice were generated as previously described [10]. Keratinocytes were isolated and cultured from adult dorsal skin in FAD medium (one part Ham’s F12, three parts Dulbecco’s modified Eagle’s medium, 1.8 × 10^–4^ M adenine), supplemented with 10% fetal calf serum (FCS) and a cocktail of 0.5 μg/mL hydrocortisone, 5 μg/mL insulin, 1 × 10^–10^ M cholera enterotoxin and 10 ng/mL epidermal growth factor (HICE cocktail) [49]. For the co-culture scRNA-seq experiment, WT and K14ΔNβ-cateninER keratinocytes were cultured on 12 well plates in a ratio of 9:1 for a total of 200,000 cells per well and allowed to attach for 24 h. Subsequently, cells were treated with 4-OHT (200nM) or DMSO as a control. After 24 h of treatment, cells were trypsinised and resuspended as a single cell suspension.

### Single cell capture, library preparation and RNA-sequencing

Single keratinocytes were captured on a medium-sized (10–17 μm) microfluidic chip (C1, Fluidigm). Cells were assessed for viability (LIVE/DEAD assay, Life Technologies) and C1 capture sites were imaged by phase contrast to determine empty and doublet capture sites. Cells were loaded onto the chip at a concentration of 300 cells μL^–1^. Doublet or non-viable cells were excluded from later analysis. Cell lysis, reverse transcription and ccomplementary DNA (cDNA) amplification were performed on the C1 Single-Cell Auto Prep IFC, as per the manufacturer’s instructions. For cDNA synthesis, the SMART-Seq v4 Ultra Low Input RNA Kit (Clontech) was used. Single cell Illumina NGS libraries were constructed with Nextera XT DNA Sample Prep kit (Illumina). Sequencing was performed on Illumina HiSeq4000 (Illumina) using 100-bp paired-end reads.

### Bulk RNA extraction and real-time qPCR

Total RNA was purified with the RNeasy mini kit (Qiagen) with on-column DNaseI digestion, according to the manufacturer’s instructions. RNA was reverse transcribed with SuperScript III (Invitrogen). PCR reactions were performed with TaqMan Fast Universal PCR Master Mix and Taqman probes purchased from Invitrogen.

### RNA-seq quantification and statistical analysis

#### Processing of reads and quality control

Reads were preprocessed using FastQC [50] and Cutadapt [51]. Sequences were aligned to the Mus Musculus genome (GRCm38) using Tophat [52] discarding multiply-mapped reads. Gene level counts were extracted using featureCounts [53]. Transcript levels were quantified as TPM. Genes with a TPM > 1 were considered as expressed. We filtered cells for analyses on the basis of number of aligned reads (> 200,000), percentage of ribosomal reads (< 2%) and number of genes expressed (> 2000). A total of 254 cells were taken forward for analysis. To assess replicate consistency, we pooled single cells within each replicate and observed that between-replicate Pearson correlation was > 0.96 for both control and non-cell autonomous beta-catenin signalling activated conditions.

### Identification of K14ΔNβ-cateninER cells

K14ΔNβ-cateninER cells were identified by aligning RNA-seq reads to the transgene locus using bowtie2 [54]. Subsequently, cell identity was confirmed using qRT-PCR with Fast SYBR Green Master Mix (ThermoFisher Scientific) using the primers ATGCTGCTGGCTGGCTATGGTCAG (forward) and ATAGATCATGGGCGGTTCAGC (reverse) spanning the beta-catenin estrogen-receptor junction.

### Dimensionality reduction, cell state map and pseudotransition

We performed dimensionality reduction and constructed the principal graph representing transitions between all possible cell states using DDRTree from Monocle2 [12]. All DDRTree dimensionality reduction was performed using default parameters and a final dimensionality of two. We initially performed this analysis on WT cells to determine the unperturbed cell state map. Subsequently we applied the DDRTree algorithm on the Wnt + cell exposed group to confirm that we independently achieve a similar cell state map. We used all 254 cells for the final transcriptomic state map and differential expression to obtain cell state marker genes. Cell clusters obtained from Monocle were confirmed by a combination of dimensionality reduction of the cells using t-distributed stochastic neighbour embedding (tSNE) [55] and cluster identification with DBSCAN [56]. Differential gene expression analysis was performed using Monocle 2 and VGAM using a likelihood ratio test controlling for batch effects and number of aligned reads per cell. Genes were filtered for log-2-fold change > 0.5 and an adjusted *p* value < 0.05. Expression profiles from this study were correlated with expression profiles from Joost et al. (single cell RNA-seq, GSE67602), Zhang et al. (bulk microarray, GSE16516) and Lien et al. (bulk microarray, GSE31028) using Pearson correlation coefficient of all genes expressed TPM > 1 in more than five cells.

### Heterogeneity analysis

Differential gene dispersion was performed using the Kolmogorov–Smirnov test after subtracting group mean expression from each group. Differentially dispersed genes were defined as q value < 0.05. We filtered for genes with a coefficient of variation (CV) fold change > 2 between the state in question and the remainder of the population. Enrichment of gene log-fold change in heterogeneity was performed using a mean-rank gene set enrichment test on GO Biological Process terms as described previously [57].

### Pseudotransition gene expression and TF enrichment

Pseudotransition cell ordering was determined by applying the Monocle pseudotime algorithm to the states with significantly different proportions of control and Wnt + exposed cells (states A, B, C and D). GO enrichment was performed on the resulting clusters of temporal gene expression using enrichR [58]. TF enrichment was performed by quantifying over-representation of target genes in the set of temporally regulated genes using the ChEA ChIP-X TF binding database [59].

### Immunofluorescence, imaging and neighbour cell quantification

#### Immunofluorescence staining

The following antibodies were used: β-catenin (1:250, Sigma); Smad4 (1:250, Sigma); Bcl3 (1:250, Sigma). For EdU experiments (Molecular Probes; C10337), half of the cell culture medium was replaced with medium containing EdU for a final concentration of 10 μM EdU 30 min before fixation. Similarly, for OPP experiments (Molecular Probes; C10456) half of the cell culture medium was replaced 30 min before fixation with medium containing OPP for a final concentration of 20 μM OPP. Cultured cells were fixed with 4% PFA for 10 min followed by permeabilisation with 0.1% Triton X-100 for 10 min at room temperature. Cells were blocked for 1 h at room temperature with 1% BSA in PBS. Primary antibody incubation was carried out for 90 min at room temperature. Samples were labelled with Alexa Fluor (488, 555, 647)- conjugated secondary antibodies for 1 h at room temperature. Cells were imaged within 24 h using an Operetta or Operetta CLS High-content Imaging System (PerkinElmer). Single cell cytoplasmic and nuclear fluorescence intensities were quantified with Harmony software (PerkinElmer) and analysed in R.

### Neighbour cell quantification

For neighbouring cell quantification K14ΔNβ-cateninER cells were labelled with CellTracker Green CMFDA dye (Molecular Probes) according to the manufacturer’s instructions. Single cell fluorescence intensity data and positional information were analysed in R. For each K14ΔNβ-cateninER CellTracker + cell the mean fluorescence intensity of neighbouring cells was calculated. Neighbouring cells were defined as the nearest cell within 20 μm (nucleus-to-nucleus distance). The mean number of neighbours was 5.4, as expected from a hexagonal packing model below confluence with mean cell diameter of 8 μm. K14ΔNβ-cateninER cells were excluded if more than two neighbouring cells were also CellTracker+. Fluorescence intensity distributions from biological and technical replicates were pooled and contrasted between conditions using the non-parametric Kolmogorov–Smirnov test.

## Additional files

Additional file 1: Figure S1. Quality control of single cell RNA-seq libraries. **Figure S2.** Comparison of heterogeneity and expression between transition states. (PDF 694 kb)

Additional file 2: Table S1. Related to Fig. 1e. List of marker genes for each keratinocyte cell state (states A–E). **Table S2.** Related to Fig. 3f. Differentially dispersed genes for states A–E. **Table S3.** Related to Fig. 4a. Genes dynamically expressed (and statistically significant) along the NCA Wnt-induced cell state transition. Cluster represents the four clusters of gene expression shown in Fig. 4a. **Table S4.** Related to Fig. 4b-d. List of TFs identified as regulating the NCA Wnt-induced cell state transition. (XLSX 71 kb)
