# Supplementary material for: Epidermal Wnt signalling regulates transcriptome heterogeneity and proliferative fate in neighbouring cells
Source: Genome Biol. 2018 Jan 15;19:3. doi: 10.1186/s13059-017-1384-y (PMC5769491; doi:10.1186/s13059-017-1384-y)
Supplement: Supplementary file 1 — Quality control of single cell RNA-seq libraries. Figure S2. Comparison of heterogeneity and expression between transition states. (PDF 694 kb) [file 13059_2017_1384_MOESM1_ESM.pdf]

Additional File 1: Supplementary figures for:

Epidermal Wnt signalling regulates transcriptome heterogeneity and proliferative fate in neighbouring cells

Arsham Ghahramani[1,2] , Giacomo Donati[2,3] , Nicholas M. Luscombe[1,4,5,†] and Fiona M. Watt[2,†]

[1]The Francis Crick Institute, 1 Midland Road, London NW1 1AT, United Kingdom
[2]King's College London, Centre for Stem Cells and Regenerative Medicine, 28th 8 Floor, Tower Wing, Guy's Hospital, Great Maze Pond, London SE1 9RT, United Kingdom
[3]Department of Life Sciences and Systems Biology, University of Turin, Via Accademia Albertina 13, 10123 Torino, Italy
[4]UCL Genetics Institute, University College London, London WC1E 6BT, United Kingdom
[5]Okinawa Institute of Science & Technology Graduate University, Okinawa 904-0495, Japan

[†] Correspondence to: fiona.watt@kcl.ac.uk and nicholas.luscombe@crick.ac.uk

Supplementary Figures

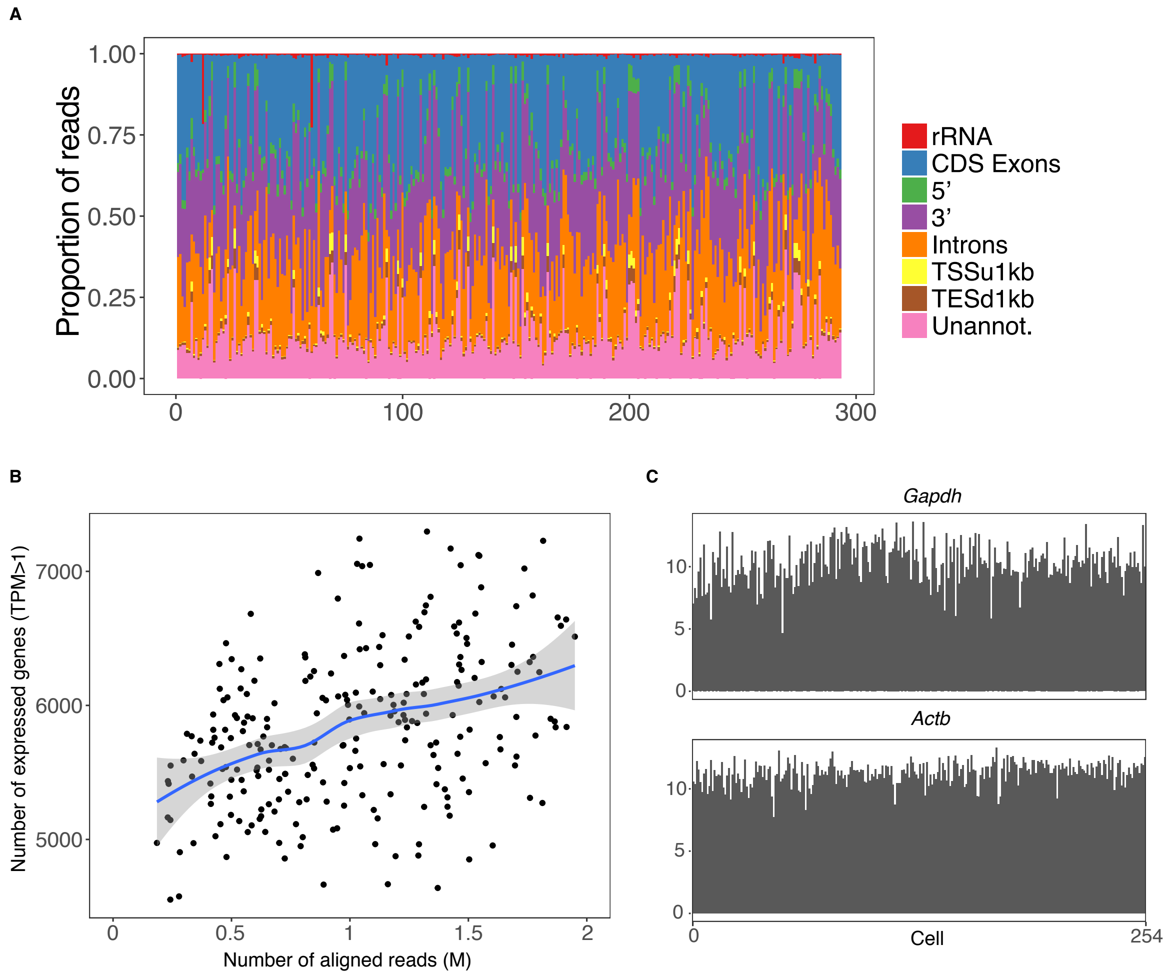

Figure S1. Quality control of single cell RNA-seq libraries

A) Stacked proportional barplot showing read alignment coverage distribution for all cells before QC filtering. TSSu1kb - region up to 1kb upstream of the transcription start site. TESd1kb - region up to 1kb downstream of the transcription end site. Unannot. - unannotated intergenic regions.

B) Scatter plot showing relation between number of aligned reads and number of genes detected as expressed (TPM > 1).

C) Barplots showing expression of two ubiquitously expressed genes (*Gapdh*, *Actb*) for 254 cells which have passed QC filtering.

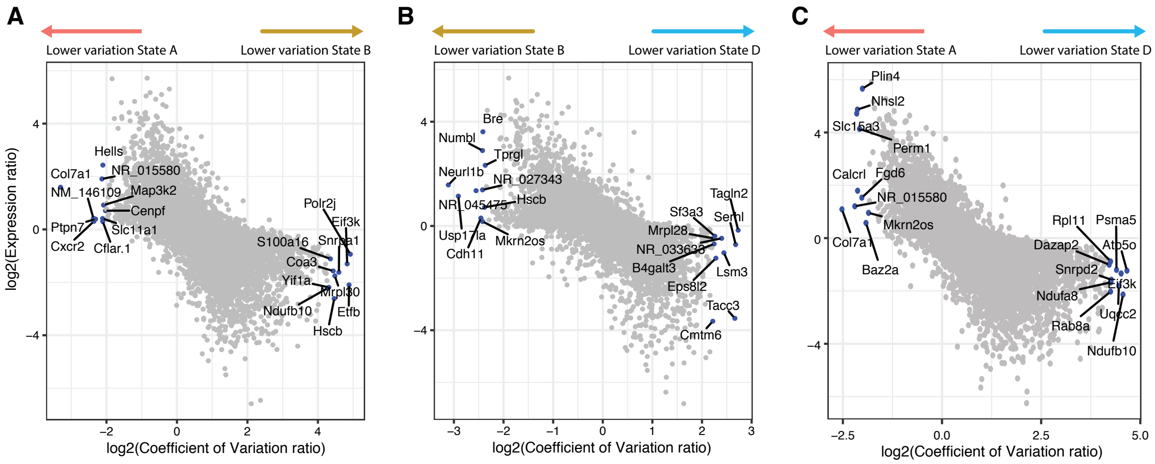

Figure S2. Comparison of heterogeneity and expression between transition states

A-C) Scatterplots showing the log-ratio of coefficient of variation versus the log-ratio of gene expression between pairs of pseudotransition states.
